# Porcine Model of Spinal Cord Injury: A Systematic Review

**DOI:** 10.1089/neur.2022.0038

**Published:** 2022-09-01

**Authors:** Carly Weber-Levine, Andrew M. Hersh, Kelly Jiang, Denis Routkevitch, Yohannes Tsehay, Alexander Perdomo-Pantoja, Brendan F. Judy, Max Kerensky, Ann Liu, Melanie Adams, Jessica Izzi, Joshua C. Doloff, Amir Manbachi, Nicholas Theodore

**Affiliations:** ^1^ Department of Neurosurgery, Johns Hopkins University School of Medicine, Baltimore, Maryland, USA.; ^2^ Department of Biomedical Engineering, Johns Hopkins University School of Medicine, Baltimore, Maryland, USA.; ^3^ Department of Molecular and Comparative Pathobiology, Johns Hopkins University School of Medicine, Baltimore, Maryland, USA.

**Keywords:** animal model, pig, porcine, SCI, spinal cord injury, swine

## Abstract

Spinal cord injury (SCI) is a devastating disease with limited effective treatment options. Animal paradigms are vital for understanding the pathogenesis of SCI and testing potential therapeutics. The porcine model of SCI is increasingly favored because of its greater similarity to humans. However, its adoption is limited by the complexities of care and range of testing parameters. Researchers need to consider swine selection, injury method, post-operative care, rehabilitation, behavioral outcomes, and histology metrics. Therefore, we systematically reviewed full-text English-language articles to evaluate study characteristics used in developing a porcine model and summarize the interventions that have been tested using this paradigm. A total of 63 studies were included, with 33 examining SCI pathogenesis and 30 testing interventions. Studies had an average sample size of 15 pigs with an average weight of 26 kg, and most used female swine with injury to the thoracic cord. Injury was most commonly induced by weight drop with compression. The porcine model is amenable to testing various interventions, including mean arterial pressure augmentation (*n* = 7), electrical stimulation (*n* = 6), stem cell therapy (*n* = 5), hypothermia (*n* = 2), biomaterials (*n* = 2), gene therapy (*n* = 2), steroids (*n* = 1), and nanoparticles (*n* = 1). It is also notable for its clinical translatability and is emerging as a valuable pre-clinical study tool. This systematic review can serve as a guideline for researchers implementing and testing the porcine SCI model.

## Introduction

Spinal cord injury (SCI) is a debilitating disease resulting in significant morbidity and mortality.^1,2^ In the United States, ∼450,000 persons are currently diagnosed with SCI, and nearly 17,000 new cases occur annually.^3^ The mainstay of treatment involves surgical decompression and aggressive hemodynamic management.^4^ However, these treatment options offer only modest improvements, and their efficacy is largely limited to mild injuries. Unfortunately, few additional treatments have received clinical approval in recent decades, and outcomes remain poor.^5–7^ Development of new therapeutic options relies on *in vitro* and animal model testing. Therefore, there is an urgent need for the development of valid animal models to elucidate our understanding of the pathogenesis of SCI and test novel treatment strategies.

Rodent models are often utilized because they are easy to handle, inexpensive, and relatively similar to humans in functional and electrophysiological outcomes.^8–10^ However, there are significant anatomical and physiological differences in cord and subarachnoid space size, corticospinal tract location, and the capacity for spontaneous recovery.^10–14^ As a result, despite multiple treatments demonstrating success in *in vivo* rodent models, they have not shown efficacy in human clinical trials.^15^

In contrast, the swine model provides a highly translational, pre-clinical paradigm for SCI.^2,16^ First, the porcine spine and spinal cord anatomy are notably similar in vertebral body height and spinal canal dimensions to those of a human.^12,17,18^ Size comparability is important for testing technologies, such as catheters and bioreactors, otherwise too large for rodent spinal canals. Second, the swine spinal vasculature is comparable to that of a human, allowing for examination of the ischemic secondary phase of SCI.^19^ Third, pigs have a similar neuronal organization of their corticospinal tract.^20^

Although the porcine SCI model is becoming increasingly popular, there is a large variety of testing parameters to consider, including differences in injury method, breed, behavioral outcomes, and histopathology. The model can therefore pose a challenge to researchers investigating SCI. Here, we systematically review the current literature on porcine models of SCI. Our primary objectives are to: 1) elucidate the array of characteristics used in developing and studying the animal model and 2) report the interventions and therapeutics that have been tested in this model.

## Methods

### Literature search

A systematic review was conducted on October 8, 2021, according to the Preferred Reporting Items for Systematic Reviews and Meta-Analyses (PRISMA) guidelines using the following databases: PubMed/MEDLINE, Embase, Cochrane Library, Scopus, Web of Science, and ClinicalTrials.gov. Full search queries are listed in Supplementary Table S1. The bibliographies of studies meeting the inclusion/exclusion criteria were also reviewed to identify additional studies.

Studies were included if they were a full-text English article utilizing a porcine SCI model and excluded if they were: 1) not available in English, 2) not a full manuscript (e.g., abstract, poster, etc.), 3) studying an animal model other than porcine, or 4) studying a pathology other than SCI.

### Data extraction

Eligible studies were independently screened against these criteria by two reviewers (A.H. and D.R.) using the Covidence systematic review application (Covidence, Melbourne, Victoria, Australia), with a third independent reviewer (C.W.L.) serving as a referee in cases of disagreement. Studies meeting the inclusion criteria then underwent data extraction by two authors (A.H. and C.W.L.) using Microsoft Excel (Microsoft Corporation, Redmond, WA). Extracted details included sample size, breed, sex, age, weight, goal, intervention, injury location, cause of injury, time to end-point, outcomes, rehabilitation measures, and conclusions.

Study quality for the articles that tested an intervention for SCI was assessed using SYRCLE's risk-of-bias tool for animal studies, an adapted version of the Cochrane risk of bias tool for randomized controlled trials (Supplementary Table S2).^21^ A judgment of yes indicates a low risk of bias, no indicates a high risk of bias, and unclear indicates insufficient detail reported.

### Institutional review board approval

Institutional review board approval was not required for this work.

## Results

**FIG. 1.**
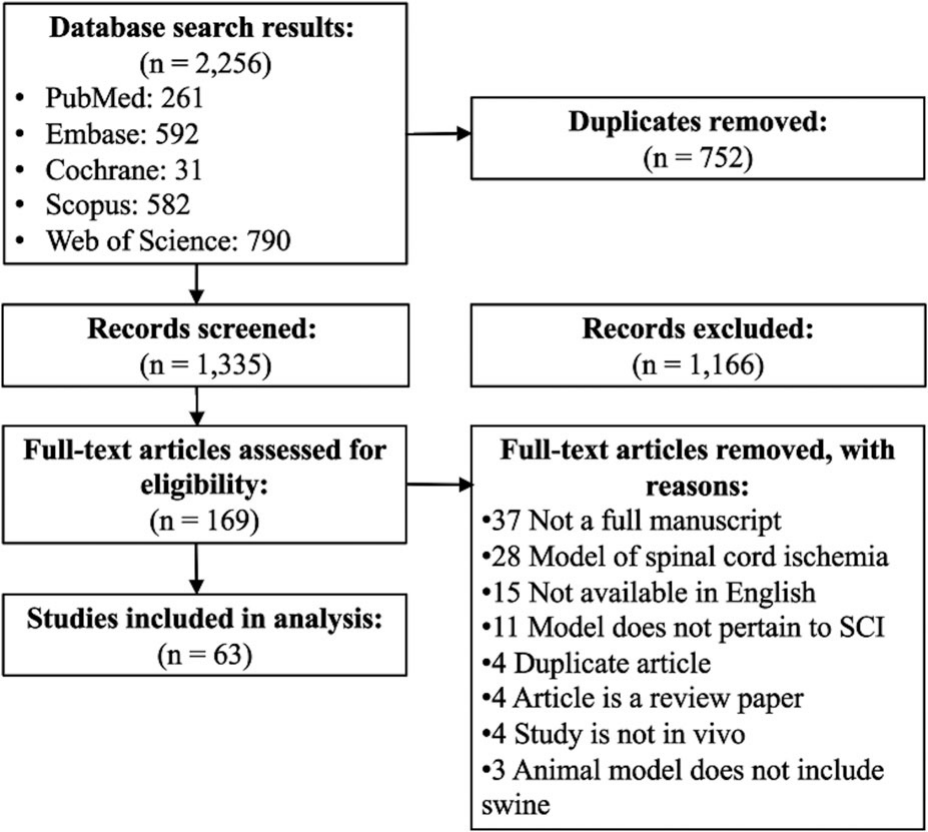
PRISMA diagram of systematic review. PRISMA, Preferred Reporting Items for Systematic Reviews and Meta-Analyses; SCI, spinal cord injury.

**FIG. 2.**
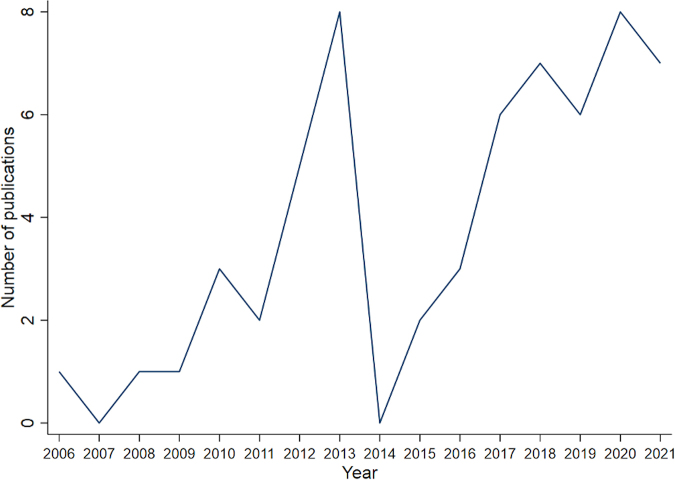
Number of publications per year utilizing the porcine SCI model included in our analysis. Three publications were published before 2005, not included above, one in 1996 and two in 1997. SCI, spinal cord injury.

We identified 1335 unique articles, of which 169 underwent full-text review, and 63 were eligible for extraction (Fig. 1). Thirty-three studies (52%) studied various aspects of the pathogenesis of SCI (Supplementary Table S3) whereas 30 studies (48%) tested an intervention on the SCI model (Supplementary Table S4). Study characteristics are summarized in Table 1. The first study describing a SCI model was published in 1996, and the number of publications has steadily increased beginning in 2005 (Fig. 2).^22^ Studies included an average of 15 animals with an average weight of 26.0 ± 11.7 kg. Forty-five studies (71%) used female swine, whereas three studies (5%) used male, three studies (5%) used both, and 12 (19%) studies did not specify. Most studies (*n* = 36, 67%) did not specify the swine age, whereas the remaining 27 were divided between those with swine ≤3 months (*n* = 6, 10%), 3–6 months (*n* = 15, 24%), 6 months to 1 year (*n* = 5, 8%), and >1 year. The most common location of injury was at the thoracic cord (*n* = 50, 80%), followed by the lumbar (*n* = 7, 11%) and cervical (*n* = 3, 5%) regions.

**Table 1.** Summary Statistics of Included Articles

Characteristic	
Sample size Weight (kg)	15 ± 11 26 ± 12
Sex	
Male Female Both Not specified	3 (5%) 45 (71%) 3 (5%) 12 (20%)
Age	
< = 1 month 1–3 months 3–6 months 6 months to 1 year >1 year Not specified	0 6 (10%) 15 (24%) 5 (8%) 1 (2%) 36 (67%)
Location	
Cervical Thoracic Lumbar Multiple levels Not specified	3 (5%) 50 (80%) 7 (11%) 2 (3%) 1 (1%)
Time to end-point	
Non-survival < = 1 day 1 day to 1 week 1 week to 1 month 1–3 months 3–6 months 6 months to 1 year Multiple time points	16 (25%) 8 (13%) 5 (8%) 2 (3%) 16 (25%) 7 (11%) 3 (5%) 6 (10%)
Injury method	
Weight drop with compression Computer-controlled impactor Weight drop Surgical clips Transection Balloon inflation Hemisection Electrocautery Gunshot Other	27 (43%) 7 (11%) 10 (16%) 4 (6%) 3 (5%) 3 (5%) 2 (3%) 2 (3%) 2 (3%) 3 (5%)
Rehabilitation (if survival surgery, *n* = 47)	
Received Did not specify	10 (21%) 37 (79%)
Interventions	
None MAP augmentation Electrical stimulation Stem cell therapy Hypothermia Biomaterials Gene therapy Steroid Nanoparticles Other	33 (52%) 7 (11%) 6 (10%) 5 (8%) 2 (3%) 2 (3%) 2 (3%) 1 (2%) 1 (2%) 4 (6%)

MAP, mean arterial pressure.

**FIG. 3.**
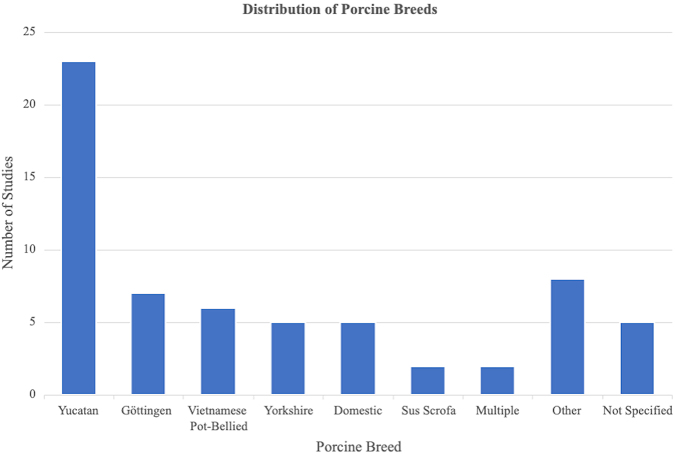
Breakdown of the different pig breeds studied in the included articles.

Six different breeds of swine were represented among the studies, including Yucatan (*n* = 23, 37%), Göttingen minipig (*n* = 7, 11%), Vietnamese Pot-bellied (*n* = 6, 10%), American Yorkshire (*n* = 5, 8%), Domestic (*n* = 5, 8%), and wild boar (Sus scrofa; *n* = 2, 3%; Fig. 3). Two studies (3%) included multiple breeds, and five studies (8%) did not specify the breed. Breeds that were only studied once in the included articles or were specified with generic terms, such as farm pig or minipig, were classified as other (*n* = 8, 13%).

Time to end-point varied among the studies, including 16 (25%) non-survival surgeries, eight (13%) maintained their pigs under a day (13%), five (8%) euthanized between 1 and 7 days, two (3%) between 1 week and 1 month, 16 (25%) between 1 and 3 months, seven (11%) between 3 and 6 months, three (5%) between 6 months and 1 year (5%), and six (10%) used multiple time points. The most common method of inducing SCI consisted of a weight drop with subsequent compression (*n* = 27, 43%), followed by computer-controlled impactor (*n* = 7, 11%) and weight drop without compression (*n* = 10 [16%]; Table 1). Of these studies, five studies used a weight ≤25 g, 32 studies used a weight between 25 and 50 g, and one study used a weight between 50 and 100 g. Twenty-one studies dropped the weight from a height between 10 and 20 cm, whereas four used a height between 30 and 40 cm, and nine chose a height between 41 and 50 cm. One study used a height exceeding 50 cm. Studies using compression kept the weight on the spinal cord anywhere between 5 and 480 min, with the average being 84 min.

Of the 47 survival surgeries, 10 (21%) described rehabilitation measures whereas 37 (79%) did not. Forty-four studies (70%) included histological analysis (Table 2)**.**

**Table 2.** Description of the Four Most Common Histology and Immunohistochemistry Stains Used on Specimens Collected from the Porcine SCI Model^1,26,44^

Methodologies	N	Function
Histology		
H&E Eriochrome cyanine R Luxol fast blue Azur-eosin	17 16 7 2	Distinguish nuclear and cytoplasmic parts Stain myelinated fibers Stain myelinated fibers Distinguish spared from injured tissue
Immunohistochemistry		
GFAP NF Iba1 NeuN	10 9 5 4	Expressed by astrocytes Found in cytoplasm of neurons Expressed in microglia Neuronal nuclear antigen

GFAP, glial fibrillary acidic protein; H&E, hematoxylin and eosin; NF, neurofilaments; Iba1, ionized calcium-binding adaptor molecule 1; NeuN, neuronal nuclear protein.

The most common interventions tested were mean arterial pressure (MAP) augmentation (*n* = 7), electrical stimulation (*n* = 6), stem cell therapy (*n* = 5), hypothermia (*n* = 2), biomaterials (*n* = 2), gene therapy (*n* = 2), steroids (*n* = 1), and nanoparticles (*n* = 1). Four studies, classified as other, included spinal shortening, vibration simulating transport, and delivery of neuroprotective factors.

### Bias assessment

Bias analysis on existing animal experimental literature is challenging because of poor reporting quality on details like randomization, housing conditions, and blinded status of assessors and caretakers. We assessed bias for studies that tested an intervention using the SYRCLE's risk-of-bias tool for animal studies. Of the 30 experimental studies, none reported information about randomization of housing, only two reported blinded investigators, and two reported baseline similarity in experimental groups. When studies reported these factors, they adequately addressed bias in the respective categories. However, for most studies, there was insufficient information to assess bias (Supplementary Table S2).

## Discussion

The porcine model is one of the most clinically relevant models for SCI. Nevertheless, the rodent model still serves as the most common animal model of SCI, likely attributable to their lower cost and ease of care.^23^ Whereas rodent models remain a useful tool in SCI research, the significant differences in anatomy and neuronal organization between rodents and humans pose a barrier to clinical translation. Therefore, the pig model is an attractive option. Pigs have a similar corticospinal tract as humans and are similar in spinal cord size and dimensions, vertebral body height, and circulatory system.^1,9,17^ Indeed, Skinnider and colleagues used proteomic analysis of cerebrospinal fluid (CSF) and serum samples to show that there is an ∼80% similarity in the molecular response to SCI between the two species.^24^ As a result, we sought to investigate the potential uses of the porcine model and summarize the various models in the literature. Overall, the included studies used a large variety of swine characteristics, study outcomes, and interventions.

### Characteristics of the selected swine

Several breeds of swine have been used in SCI models. The choice of breed reflects features such as gentle nature, space limitations, lab familiarity, and species availability. Swine weighed an average of 26 kg, highlighting the trade-off between matching a typical human weight and allowing for space limitations, ease of post-operative care, and mobility in a research setting. Nevertheless, pigs can attain body weights similar to that of humans in a significantly less amount of time, and these weights bear a greater degree of similarity to humans compared to other typical SCI animal models, such as rodents. For studies focusing on the pediatric population, the pig weight can be matched well to that expected in a human child. Additionally, most studies used female swine, likely because of their smaller size, gentle nature, and ease of urinary catheter insertion.^25^

### Method of injury

The injury method is critical to the translational potential of a SCI model. The most common form of injury identified in the systematic review used a weight drop followed by compression. This is designed to mimic the typical sequelae of SCI, wherein the initial mechanical injury is followed by swelling of the spinal cord parenchyma, resulting in compression by the surrounding dura and bony spine.^23,26,27^ A diverse range of compression length times were used to produce injuries of different severities, similarly reflecting the ranges of time between injury and surgical decompression in humans. For example, the STASCIS (Surgical Trial of Acute Spinal Cord Injury Study) found two cohorts representing the average time between injury and surgical decompression—an early group of 14.2 ± 5.4 h and a late group of 48.3 ± 29.3 h.^28^ Naturally, simulating such extended compression lengths is logistically challenging in a research setting because of limitations in anesthesia and surgical manpower, such that the maximum length identified was 8 h.^11,29,30^

**FIG. 4.**
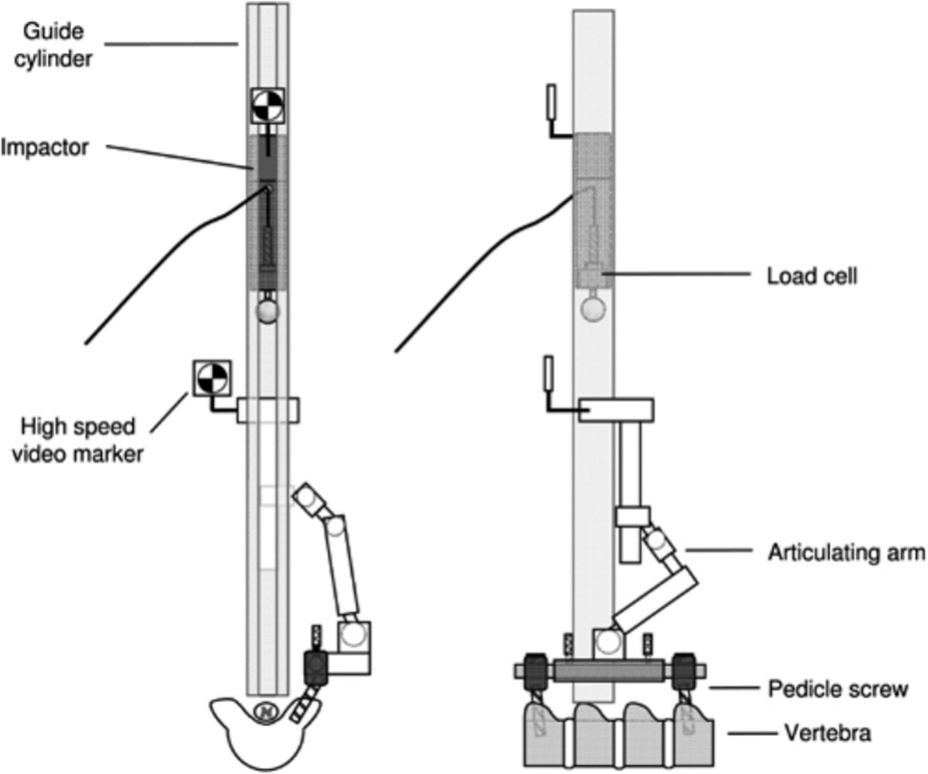
An example of a weight-drop apparatus published by Jones and colleagues in which pedicle screws are used to secure the articulating arm that connects to the guide cylinder that the impactor travels through.^12^ (Reprinted from Jones CF, Lee JH, Kwon BK, Cripton PA. Development of a large-animal model to measure dynamic cerebrospinal fluid pressure during spinal cord injury: Laboratory investigation. J Neurosurg Spine. 2012;16(6):624–635, with permission from the Journal of Neurosurgery Publishing Group.)

A major limitation of the weight-drop method is inconsistencies in the severity of injury across subjects with the same amount of weight dropped. As a result, some studies use a paralytic agent before injury to avoid animal arousal or uncontrolled movements during injury.^26^ Researchers may also suppress mechanical ventilation during the weight drop to avoid respiratory motion,^10,31^ and pedicle screws are frequently used to secure the weight-drop apparatus (Fig. 4**)**.^8,11^ A weight-drop apparatus can also be equipped with multi-modal sensors, including an acceleration sensor, load cell, and photodetector, to record impact parameters such as impact velocity, impulse force, and maximally compressed displacement.^10,16^ To prevent the spinal cord from undergoing lateral displacement during impact, a device consisting of bilateral metal plates can be used to entrap the spinal cord on both sides.^10^ Fusing adjacent vertebrae can prevent vertebral bodies from yielding during impact.^10,26^ Even with these techniques, variations in injury severity frequently result using weight-drop impactors. Other methods, such as a spring-loaded impactor, have been shown to produce more consistent and reproducible impacts.^10,26^

Other methods of injury include gunshots, vertebral column shortening, iatrogenic trauma, or electrocautery. The gunshot model is useful for studying war-related SCI.^22,32^ Modi and colleagues tested the amount of vertebral column shortening leading to a loss of motor-evoked potentials (MEPs), which corresponded to the average vertebral body height at the thoracolumbar level.^33^ Montes and colleagues simulated SCI from displaced pedicle screws by sequentially compressing the swine spinal cord with metal sticks on both sides.^34^ Finally, Skinner and colleagues examined SCI by performing electrocautery at the dural root sleeves within 6–8 mm of the spinal cord, finding that 20 sec of electrocautery with a temperature of at least 57°C can induce MEP loss.^35^ These studies allow us to better understand causes of SCI other than the more common mechanical trauma followed by compression.

Injury models frequently produce complete paraplegia, although several studies focused on generating incomplete SCI. For example, del Cerro and colleagues performed hemisection with a 1-cm excision of neural tissue to induce monoparesis of the forelimb.^17^ Given the large number of injuries classified as American Spinal Injury Association Grades B–E, indicating incomplete injury, animal models that can appropriately mimic such injuries are particularly important.

Minimally invasive models have recently been designed to replace the typical laminectomy required to induce injury.^36^ Foditsch and colleagues used a guidewire and balloon under computed tomography (CT) guidance to compress the epidural space and spinal cord at T12, requiring around only 1 h, including 30 min of compression time.^1^ Other groups have begun implementing this method.^27,36^ However, such models are less similar to the traumatic injury mechanisms that classically occur in humans.

Finally, some studies have investigated other factors that could exacerbate injury. Streijger and colleagues found that vibration of the injured spinal cord, mimicking those generated during transport in ground or airborne vehicles, does not worsen the histological or functional outcomes post-SCI.^37,38^ Exploring these other factors that could affect the severity of injury are vital for keeping patients as safe as possible.

### Animal care and rehabilitation

Standard protocol calls for acclimating the pigs to the research facility for at least 1 week before injury.^17^ The level of required post-operative care depends on the injury severity. Researchers should consider the animal's ability to access food and water after injury, as well as the likelihood of urinary retention or pressure sores, two major sources of morbidity and mortality.^39^ Assistance in eating and drinking must be provided until animals have sufficient mobility to access food on their own. Pressure sores can be avoided by frequently rotating the position of the animal and providing a soft, padded surface.^17,23^ In the event of urinary retention, pigs should receive a urinary catheter to prevent bladder rupture.^5,9,23,39–41^ Additionally, antibiotics and pain medication must be provided post-operatively under the guidance of a veterinarian team.^1,7^ Research teams should plan on having personnel that can regularly check on the animals, often multiple times daily in the early phase of a severe injury model.

**FIG. 5.**
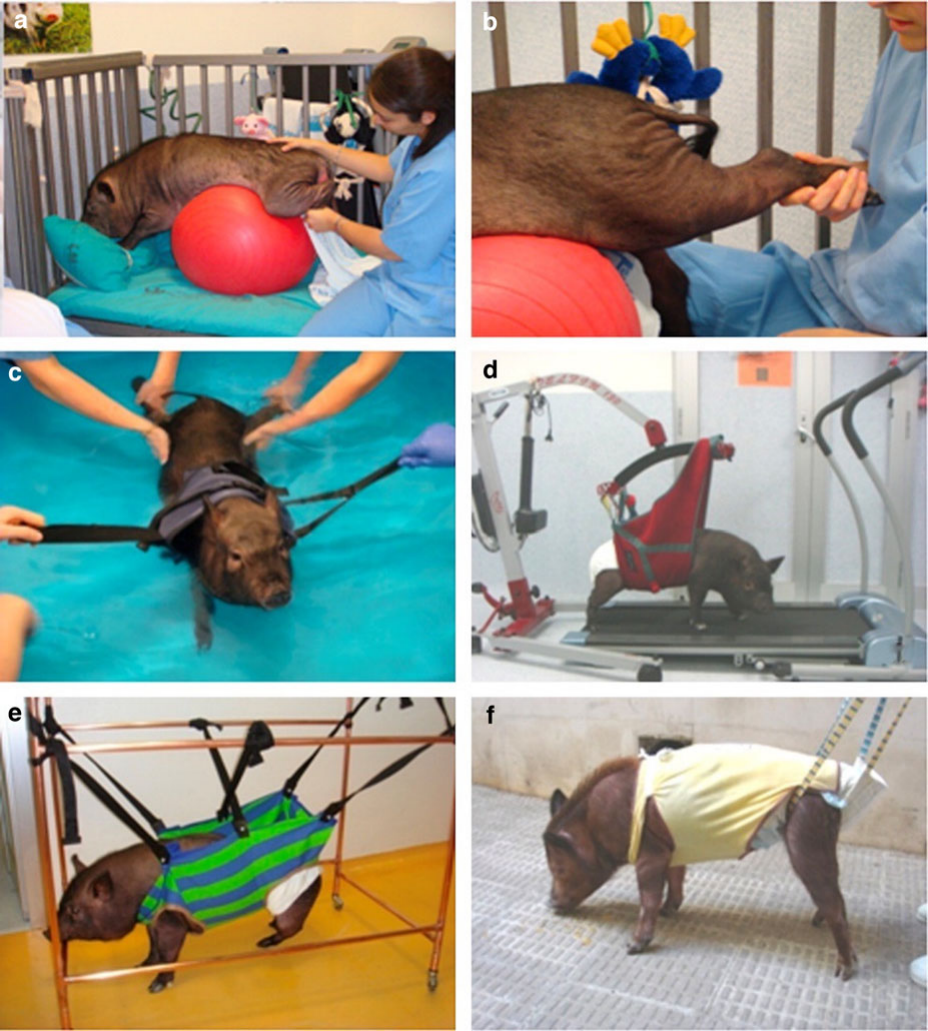
Examples of rehabilitation measures described by Zurita and colleagues. (**A,B**) Kinesitherapy with a therapeutic ball. (**C**) Hydrotherapy. (**D**) Weight-assisted treadmill. (**E,F**) Weight-assisted walking.^23^ (Reprinted from Zurita M, Aguayo C, Bonilla C, et al. The pig model of chronic paraplegia: a challenge for experimental studies in spinal cord injury. Prog Neurobiol. 2012;97(3):288–303, with permission from Elsevier.)

Rehabilitation is important post-operatively so that patients can practice navigating surroundings with their disability and relearning critical movements. Many of the included studies similarly incorporated rehabilitation into their post-injury care (Fig. 5). Rehabilitation can reduce stress in animals and facilitate the acquisition of improved functional results.^23^ Wheelchairs can be used to support the animal's body weight while they move their functional limbs to prevent muscle atrophy and reduce the development of pressure sores.^23,40^ A weight-supported–assisted treadmill is also common to promote movement relearning and muscle strengthening.^17^ Passive mobilization of hindlimbs can be used as a simpler means of rehabilitation.^23,41^ Hydrotherapy can allow for more mobility and muscle engagement, although this can be difficult to implement in laboratories.^23^ These various rehabilitation measures are often done several times weekly for the duration of the study.

### Behavioral outcomes

Behavioral outcomes are critical in tracking functional recovery after SCI and determining the efficacy of different interventions. The most common behavioral outcome used is the Porcine Thoracic Injury Behavior Scale (PTIBS), introduced by Lee and colleagues in 2013.^5,7,8,16,24,25,37,38,42–48^ This 10-point scale can be used to classify the motor function of the hindlimbs (Table 3). The PTIBS strongly correlates with less tissue damage through the epicenter of injury.^8^

**Table 3.** The 10 Points Described in the PTIBS Motor Scoring System^8^

Score	Description
1	No active hindlimb movements, with rump and knees on the ground
2	Active hindlimb movements, with rump and knees on the ground
3	Active hindlimb movements, with “weight-bearing extensions” that lift the rump and knees transiently off the ground
4	Active rhythmic hindlimb crawling with at least three reciprocating gait cycles
5	The animal can take between two and six steps with the rump and knees constantly off the ground during steps. Knees do not fully extend. Dorsal and plantar hoof placement. Impaired balance.
6	The animal can take more than six steps with the rump and knees constantly off the ground. knees do not full extend. Dorsal and plantar hoof placement. Impaired balance.
7	The animal can take two to six steps with the knees fully extended. Dorsal and plantar hoof placement. Impaired balance.
8	The animal can take more than six steps with the knees fully extended. Dorsal and plantar hoof placement. Impaired balance.
9	The animal can take more than six steps with the knees fully extended. Plantar hoof placement. Imbalanced trunk.
10	The animal demonstrates normal ambulation with normal balance.

PTIBS, Porcine Thoracic Injury Behavior Scale.

The Porcine Neurological Motor Score is also frequently used.^1,20^ This is a 14-point scoring system that assesses movement in all three joints in the lower extremities and tail with no weight support and the degree of recovery of ambulatory function with weight support.

Some studies designed their own scoring systems for motor assessment. Del Cerro and colleagues developed the Individual Limb Motor Scale and Quadruped Position Global Scale. The former scores the movement, positioning, and weight-bearing ability of each leg, whereas the latter scores the axial muscle tone, evaluated by the capacity to acquire a prone position or stand up.^17^ Gedrova and colleagues created a 20-point scale where 1–8 represent slight or extensive movement for each individual joint of the hindlimbs, 9–11 represent sweeping and the ability to get up by itself (though without the ability to maintain balance), and 12–20 characterize the ability to walk a few steps without forelimb/hindlimb coordination to consistent stepping.^49,50^ Santamaria and colleagues developed the Miami Porcine Walking Scale, a 10-point scale based on hindlimb stepping and weight bearing with 1 corresponding to complete paraplegia and 10 indicating no apparent deficit.^9,26^ The Tarlov scale, used by Zurita and colleagues, is a similar 10-point scale of hindlimb motor function.^23,41,51^ Other studies used scoring systems that are used in a clinical setting. Huang and colleagues used the modified Tarlov score, which is a shorter, 4-point scale with 0 representing no locomotion and 4 indicating normal gait.^52,53^

Kinematics can also be used to record functional outcomes, whereby markers are placed on anatomical landmarks and animal motion is captured by cameras. Common anatomical landmarks include the trochanter of the femur, knee, ankle joint, and hoof.^7,17,38,44^ Video recordings are subsequently analyzed to determine gait.

Whereas most studies evaluate motor function, some include measurements of sensory function. Withdrawal response to a mechanical stimulus, often compression of the toes with forceps, can be graded as absent or present. Some studies also perform a perianal pinch and check for any resulting vocalization.^1,20,31^

Behavioral assessment is typically performed at least weekly. Multiple independent observers are helpful in reducing individual rater bias. Overall, the sheer variety of potential behavioral outcomes and scoring systems used makes it challenging to compare the results of studies.

### Histological and immunohistochemistry outcomes

Histological analysis is often used to analyze the cellular response to SCI (Table 2). Hematoxylin and eosin (H&E) is the most common stain and can depict general morphology and the degree of tissue damage.^27,33,34,43,53,54^ Eriochrome cyanine R, commonly used in conjunction with the counterstain cresyl violet or Neutral Red, can help delineate and measure areas of white and gray matter, allowing for analysis of lesion size and neuronal degeneration.^11,25,37,38,42,45,47,48,55–57^ Luxol fast blue can also be used with cresyl violet counterstain to identify myelinated fibers in white matter and the residual preserved tissue.^10,40,49^ More severe injuries are generally associated with a lesser degree of white and gray matter sparing.^11,25,45^

Similarly, immunohistochemistry is particularly helpful in capturing the pathogenesis of SCI by analyzing changes in protein expression.^58^ The four most common proteins studied include glial fibrillary acidic protein (GFAP), neurofilaments (NF), ionized calcium-binding adaptor molecule (Iba1), and neuronal nuclear protein (NeuN)^1,2,5,8,41^ (Table 2). Other proteins of interest include Olig-2, expressed by oligodendrocytes, and Hsp27 and caspase3, which are used to evaluate the survivability of spinal cord cells.^1,40,43,44^ DAPI can be used for nuclear counterstaining.^44^ More severe injuries are generally associated with increases in GFAP, Iba1, and caspase3, and decreases in NF, NeuN, and Olig-2.^8,24,44^ These changes develop as injured axons degenerate and lose myelin, while intense infiltration and activation of microglia occurs.^20^

### Probes and sensors

Microdialysis probes can sample CSF to test for mediators of neurological injury, such as PGE_2_, glutamate, and citrulline, as well as metabolites, including lactate, pyruvate, glucose, and glycerol.^48,55–57,59,60^ Intraparenchymal sensors can be used alongside microdialysis probes to analyze spinal cord blood flow (SCBF), partial pressure of oxygen, hydrostatic pressure, and metabolic changes *in vivo*.^48,55–57,60–62^ Studies using these sensors have shown that lactate and pyruvate increase at the injury site whereas glucose remains stable. Interestingly, when contusion is followed by compression, the lactate/pyruvate ratio, glutamate, and glycerol rise whereas glucose levels fall.^55–57^ SCBF decreases immediately after SCI to 60% of baseline but increases over subsequent days, ultimately reaching 150% of baseline value.^57^ These studies show that metabolic stress and insufficient oxygen levels can lead to ischemia and hypoxia after injury.^57^

Subdural fiber-optic pressure transducers, as tested by Jones and colleagues, have been used to show a cranial-caudal distribution in pressure near the injury site, with greater increases in pressure from more severe injuries.^11,12,29,30^ Less invasive versions have been designed, including an optical epidural sensor based on near-infrared spectroscopy to monitor spinal cord oxygenation and hemodynamics in real time.^4,62^ An epidural laser Doppler flow-monitoring probe can similarly be used to measure blood flow.^27,33,60,63^ Sarwahi and colleagues found that changes in perfusion precede MEP loss by an average of 15.8 min, suggesting that SCBF can be a leading indicator of injury. These non-invasive probes are particularly useful in translating these technologies to clinical studies.^27^ These studies will allow us to better understand the pathogenesis of injury *in vivo*, while also aiding in the discovery of biomarkers that can provide more accurate prognosis.

### Electrophysiology

Electrophysiology, consisting of electromyography (EMG), MEPs, and somatosensory-evoked potentials (SSEPs), can be used as a functional analogue to assess the integrity of the spinal cord.^10^ For EMG and MEPs, the soleus, extensor carpi radialis, gluteobiceps, and tibialis anterior muscles are frequently studied.^5,7,23,25,27,33,35,44,46,64^ Signals can subsequently be analyzed for H-reflex, M-response, latency, amplitude, and duration. For SSEPs, common stimulating electrode placement includes the sural, tibial, sciatic, and median nerve.^5,10,23,25,40,41,43,46,51^ Changes in EMGs may anticipate MEP loss, whereas changes in MEPs are generally detectable before changes in SSEPs.^25,33,35,64^ Electrophysiology can be a useful objective tool for studying motor and sensory pathways to gauge injury severity.^34^

### Imaging

**FIG. 6.**
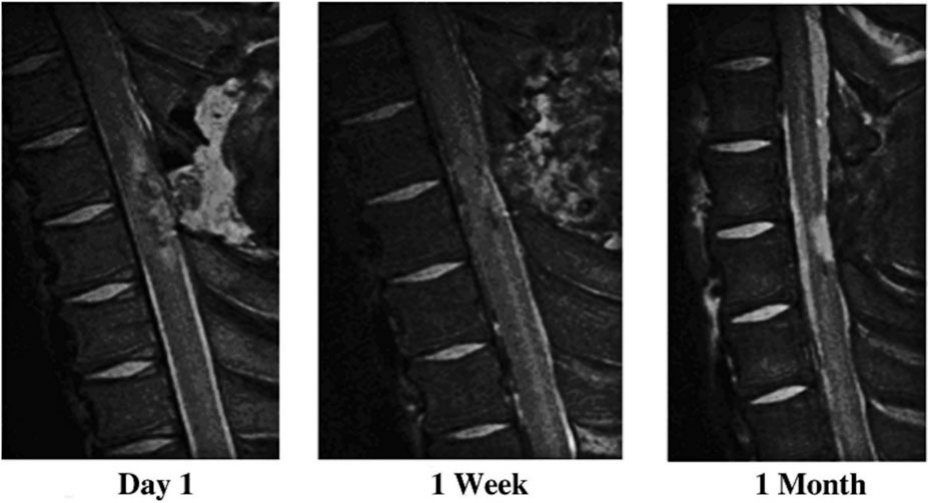
T2-weighted MRI images of an incomplete SCI in an infant piglet over 1 month demonstrating the development of necrotic tissue.^40^ MRI, magnetic resonance imaging; SCI, spinal cord injury. (Reprinted from Kuluz J, Samdani A, Benglis D, et al. Pediatric spinal cord injury in infant piglets: description of a new large animal model and review of the literature. J Spinal Cord Med. 2010;33(1):43–57, with permission from Taylor & Francis.)

Imaging, such as magnetic resonance imaging (MRI), CT, and ultrasound, can aid in the analysis of injury site or lesion dimensions.^1,9,27,40,41,48^ Kuluz and colleagues used MRI to determine the volume of necrotic tissue in a piglet model of pediatric SCI (Fig. 6).^40^ Post-mortem MRI can be used to evaluate the extent of injury and amount of spared tissue.^9,10,20,26,47,65^ MRI images may show a hypointense region on T1 and hyperintense lesion on T2, suggesting demyelination or gliosis as the cavity develops.^23,41^ Additionally, MRI can show the decrease in white and gray matter that occurs after injury.^10^

Ultrasound is another powerful imaging tool to assess the effects of SCI. Contrast-enhanced ultrasound and color Doppler have shown hypoperfusion at the injury epicenter with concomitant hyperperfusion in the adjacent regions, demonstrating the ischemia that occurs at the injury site.^9,53^ Jones and colleagues used B-mode ultrasound to demonstrate the gradual and immediate swelling that occurs after moderate and severe SCI, respectively, suggesting the need for decompression after injury.^29^ Kim and colleagues and Santamaria and colleagues used B-mode ultrasound to show that the thickness of the CSF layer is a major determinant of injury severity.^9,42^ Notably, differences in the thickness of the subarachnoid space can affect the reproducibility of an injury model.

### Serum and cerebrospinal fluid samples

Serum and CSF samples can be obtained to analyze inflammatory markers and cytokines. These include granulocyte-macrophage colony-stimulating factor (GM-CSF), interleukin (IL)-1ra, IL-2, IL-6, IL-10, IL-12, IL-18, tumor necrosis factor-alpha (TNFα), interferon-gamma, IL-1β, IL-1α, IL-4, and IL-8. GM-CSF, IL-4, IL-10, and IL-12 are generally considered to have neuroprotective potential. Meanwhile, IL-1 and TNFα may cause neuronal cell death.^5,37,44,46^ Changes in gene expression can also be analyzed through reverse-transcription polymerase chain reaction.^44^

MicroRNAs (miRNAs) and proteins in serum and CSF samples can be examined as potential biomarkers of injury. Tigchelaar and colleagues sequenced serum and CSF miRNAs collected before injury and 1, 3, and 5 days post-injury, finding that miRNA expression after injury strongly correlated with injury severity, amount of spared tissue, and functional outcomes.^45^ Therefore, miRNA levels could serve as potential biomarkers of the integrity of the spinal cord at the injury site and predict behavioral outcomes. Skinnider and colleagues used proteomic analysis of 111 human and 43 porcine samples to determine that GFAP could serve as a biochemical outcome measure of SCI severity and recovery.^24^ GFAP levels in serum have been used by other researchers as a marker of injury severity and treatment response as well.^38^

### Additional outcomes

Damage to the spinal cord can lead to major hemodynamic changes, cardiorespiratory failure, and withdrawal of sympathetic tone. Nearly 50–90% of patients with cervical SCI require hemodynamic support in the form of intravascular volume and augmentation of blood pressure. Zahra and colleagues found that immediate tachycardia and hypotension lasted for 1 and 4 h, respectively, after cervical or upper thoracic injury. These changes were accompanied by an increase in serum vasopressin and a decrease in MAP and systemic and pulmonary vascular resistance.^65^ West and colleagues similarly found that systolic blood pressure, MAP, and heart rate increased immediately after injury, although blood pressure subsequently decreased below pre-injury levels. They also found lower levels of noradrenaline and higher levels of aldosterone and angiotensin II.^39^ These studies support the current recommendations of maintaining MAP above 85–90 mm Hg for up to 2 weeks through intravascular volume loading and vasopressors.^60,66,67^ Appropriate cardiovascular management is critical to maintaining adequate spinal cord perfusion post-injury.

Other pathophysiological changes in the spinal cord have been explored *in vivo*. Akino and colleagues used phosphorus-31 nuclear magnetic resonance spectroscopy to examine changes in phosphocreatine (PCr), adenosine triphosphate (ATP), inorganic phosphate (Pi), intracellular pH (pHi), and free magnesium post-injury.^68^ They found that injury led to a reduction in ATP, PCr, and pHi and an increase in Pi.

Finally, the porcine model can be used to study the systemic effects of SCI on the body. For example, up to 84% of persons with SCI will exhibit bladder dysfunction.^69^ Using a porcine model of complete spinal cord transection, Foditsch and colleagues showed that SCI reduces contractile and elastic properties and changes the protein composition of the bladder wall.^70^ Keung and colleagues similarly found that bladders after SCI were heavier, thicker, and less contractile.^71^ These studies are important for elucidating other major causes of morbidity and mortality post-SCI.

### Interventions

The porcine SCI model is a powerful way to test potential interventions and therapeutics for SCI, including steroids, hypothermia, electrical stimulation, gene therapy, and regenerative medicine. Present guidelines for acute SCI recommend augmenting MAP to 85–90 mm Hg for the first 7 days post-injury.^30,56,57,60–63,65,66^ The porcine model has been used to test the efficacy of vasopressors, including norepinephrine, phenylephrine, and dopamine.^27,56,60,61,63^ These studies generally support the benefits of MAP augmentation to improve SCBF and clinical outcomes.^61^ Interestingly, Streijger and colleagues found that norepinephrine resulted in increased spinal cord blood perfusion and oxygenation, whereas phenylephrine resulted in decreased levels. Additionally, administering phenylephrine resulted in greater hemorrhaging, suggesting that norepinephrine might be the preferred vasopressor.^56^ Martirosyan and colleagues found that MAP augmentation led to a transient 22% increase in SCBF, and that the combination of vasopressors with CSF drainage led to a sustained 32% increase in blood flow.^63^ However, CSF drainage alone did not lead to improvements in blood flow, a finding that has been correlated in human studies.^63,72^

Importantly, some studies found negative impacts of vasopressor usage, including hemorrhage development and increases in intrathecal pressure.^61^ Additionally, other studies have found that β-agonists, such as dobutamine, may be more effective than vasopressors at improving spinal cord oxygenation and blood flow while mitigating hemorrhage.^60^

Another standard of care for SCI is surgical decompression, which involves the realignment of the spinal column and removal of bone that is compressing the spinal cord. It remains unclear whether bony decompression is sufficient to appropriately relieve the swelling cord, or whether intradural decompression by durotomy is also required. Streijger and colleagues tested the efficacy of durotomy with duraplasty and found no improvement in functional outcomes nor any differences in SCBF or oxygenation.^48^ Therefore, though surgical decompression remains an important treatment for SCI, the role of duraplasty remains unclear.

Steroids have been commonly tested as a therapeutic for reducing inflammation in SCI.^59^ However, several human clinical studies have failed to demonstrate a neurological benefit of steroid therapy, whereas others have shown only modest improvements at best. Animal studies are similarly inconclusive. For example, Bernards and colleagues tested intravenous and intrathecal methylprednisolone 30 min after injury and found no difference in *in vivo* markers of neurological injury.^59^ However, the potential role of long-term chronic steroid therapy is unclear.

Hypothermia has been explored as another anti-inflammatory tool by reducing metabolic consumption, energy demands, local edema, and oxidative stress.^49,50^ Once again, studies on the porcine model have shown mixed efficacy. Gedrova and colleagues found that local hypothermia improved hindlimb recovery in mild SCI, but not in severe injuries.^49^ Zavodska and colleagues found that hypothermia with or without a durotomy led to no significant improvements in neurological status, although hypothermia after durotomy improved white matter integrity and led to regeneration of neurofilaments.^50^ Additional studies are needed to further evaluate any potential therapeutic efficacy of hypothermia.

Electrical stimulation has been studied as a complementary approach for rehabilitation of SCI patients, supported by the hypothesis that it can retrain the intrinsic activity of the spinal cord circuitry. Hachmann and colleagues developed a protocol for wireless control of intraspinal microstimulation in the porcine SCI model to produce contraction at the hip, knee, and ankle muscles.^6^ Fadeev and colleagues tested epidural electrical stimulation with motor rehabilitation on a treadmill and found that stimulation led to behavioral, electrophysiological, and joint kinematic improvements.^7^ Islamov and colleagues added triple-gene therapy consisting of vascular endothelial growth factor, glial cell-line–derived neurotrophic factor, and nerve cell adhesion molecule, and found that animals that received both the gene therapy and stimulation experienced greater improvements in locomotor performance.^44^ Further, Keller and colleagues used sacral and pudendal neuromodulation to improve lower urinary tract function after injury,^36^ whereas Kowalski and colleagues used epidural electrical stimulation to restore an effective cough mechanism.^54^ Solis and colleagues used intermittent electrical stimulation to loaded muscles to prevent the formation of pressure-related deep tissue injury after SCI.^73^ Overall, these studies support the use of electrical stimulation as an effective tool in promoting recovery after SCI.

Nanoparticles and gene therapy can serve as a potential avenue for localized delivery or production of therapeutics. Gao and colleagues found that intravenous nanoparticles can localize to the lesion site in a contusion model,^74^ suggesting that breakdown of the blood–spinal cord barrier after injury can be used to localize the delivery of nanoparticles or drugs to the lesion site. Islamov and colleagues intrathecally injected a triple-gene therapy 10 days after SCI and found that treated animals had significant histological, electrophysiological, and clinical improvements.^43^ To avoid the need of donor cells, Islamov and colleagues next used a gene-modified leucoconcentrate prepared from peripheral blood to evaluate the possibility for personalized cell-mediated gene therapy.^75^

Delivery of neuroprotective factors, such as magnesium, has also been studied as a potential therapeutic, albeit with limited success. Streijger and colleagues tested magnesium chloride within a polyethylene glycol formulation; however, this did not result in any improvements in locomotor recovery, tissue integrity, or white or gray matter sparing.^38^

Finally, regenerative medicine through the delivery of stem cells or biomaterial scaffolds has been explored to promote healing after SCI. Lim and colleagues developed a minipig model for delivery of neural stem cells to the SCI lesion, confirming at 4 weeks the survival of the stem cells and their subsequent differentiation into glial and neuronal lineages.^2^ Mukhamedshina and colleagues found that adipose tissue mesenchymal stem cells could partially restore somatosensory signal pathways, reduce post-traumatic cavitation, and modulate astroglia activation. Shulman and colleagues used a fibrin matrix filled with peripheral blood mononuclear cells to improve tissue integrity and conduction along the posterior columns, though without showing an improvement in functional outcomes.^46^ Zurita and colleagues injected bone marrow stromal cells into the lesion and perilesional intrathecal zones and showed progressive functional recovery, detected by a return of SSEPs, over the course of several months.^41,51^ These studies illustrate the potential of regenerative medicine in the treatment of SCI and the centrality of the porcine model for pre-clinical testing.

### Limitations

Our study has several limitations. The variation in injury models and outcome methods precluded a quantitative meta-analysis. Accordingly, we cannot provide detailed guidelines on methods to produce different injury levels; however, modulating the weight or force of impact, height of drop, and compression time can all be performed in initial studies to obtain a suitable injury model. Moreover, many of the studies use small sample sizes, illustrating the need for more extensive testing. Additionally, we excluded several related pathologies such as spinal cord ischemia.

### Recommendations for developing a porcine spinal cord injury model

**FIG. 7.**
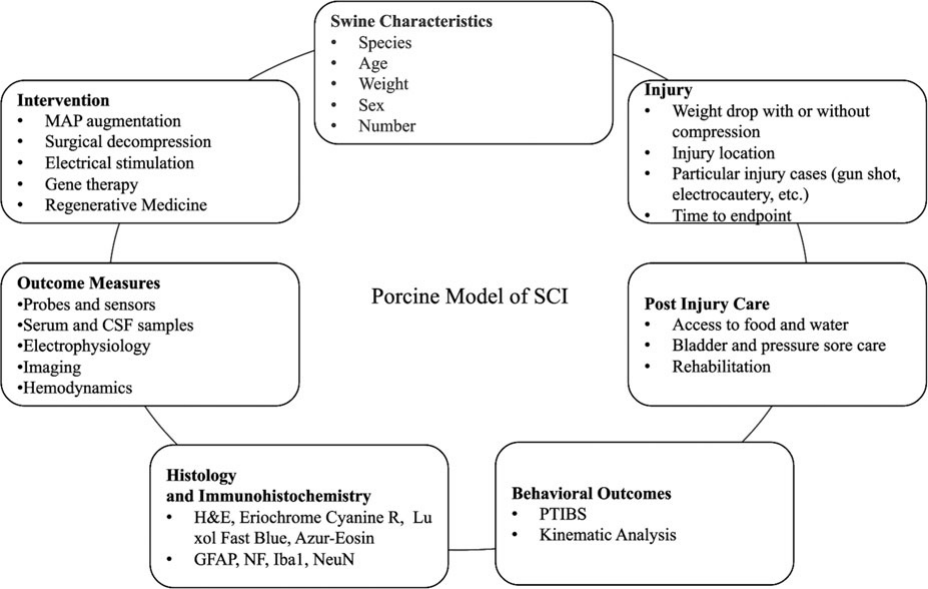
Summary of the aspects that make up the porcine model of SCI. CSF, cerebrospinal fluid; GFAP, glial fibrillary acidic protein; H&E, hematoxylin and eosin; Iba1, ionized calcium-binding adaptor molecule 1; MAP, mean arterial pressure; NeuN, neuronal nuclear protein; NF, neurofilaments; PTIBS, Porcine Thoracic Injury Behavior Scale; SCI, spinal cord injury.

Our systematic review shows that there are many important aspects to consider when developing a porcine SCI model (Fig. 7). Swine characteristics, including species, age, weight, and sex, are at the lab's discretion, though female sex is generally preferable because of their gentler nature and the feasibility of inserting a urinary catheter. Animals should be brought into the facility at least a week before surgery to allow for appropriate acclimation time. When choosing an injury method, weight drop with compression is the most common and similar to that of human injuries. Other injury methods, such as gunshots or balloon compression, can be used for testing certain injury cases or for a less invasive, faster method. After injury, post-injury care is of the utmost importance. Animals should be closely watched for their ability to access food and water, as well as the development of any pressure sores or incontinence. Having a veterinarian team available is critical for ensuring safety of the pig during this stage. Pain medication and antibiotics should be given regularly under the guidance of the veterinarian team.

Rehabilitation can also be incorporated at this point, which can improve recovery outcomes and overall animal well-being. Behavioral measures, especially the PTIBS or kinematic analysis, can be performed pre- and post-operatively weekly for the duration of the study to track and analyze functional recovery. Many different outcome measures can be used depending on the study goals. Probes and sensors can be used to test CSF or the parenchyma for blood flow, oxygenation, or metabolites. Serum and CSF samples can be taken to look at inflammatory markers or discover new biomarkers of injury. Electrophysiology can be used as a functional correlate, whereas imaging can capture the lesion dimensions and tissue involvement. Finally, the ultimate purpose of the SCI model is to develop better treatments and interventions. The model has shown efficacy of MAP augmentation, surgical decompression, electrical stimulation, gene therapy, and regenerative medicine. Other interventions, including steroids and hypothermia, have not yet been validated by the animal model. Incorporating and considering the factors described above will allow for an easier and more successful animal model.

## Conclusion

The porcine model of SCI is notable for its translational relevance to humans. However, the preponderance of injury methods and behavioral outcomes, along with logistical complexities in post-operative care and rehabilitation, have posed obstacles to the widespread adoption of this model. Here, we conducted a systematic review to summarize the available literature on the porcine model of SCI. We found 63 studies, with 33 using the animal model to investigate the pathogenesis of SCI and 30 studies testing an intervention or therapeutic for SCI. We summarized the methods to generate the injury and provide post-operative care and described behavioral outcomes, imaging techniques, histology, immunohistochemistry, and interventions that have been tested (Fig. 7). This review can serve as a guide in the development of a porcine model of SCI.

## Supplementary Material

Supplemental data

Supplemental data

Supplemental data

Supplemental data

## Abbreviations Used

ATP: adenosine triphosphate
CSF: cerebrospinal fluid
CT: computed tomography
EMG: electromyography
GFAP: glial fibrillary acidic protein
GM-CSF: granulocyte-macrophage colony-stimulating factor
H&E: hematoxylin and eosin
Iba1: ionized calcium-binding adaptor molecule
IL: interleukin
MAP: mean arterial pressure
MEP: motor evoked potential
miRNA: microRNA
MRI: magnetic resonance imaging
NeuN: neuronal nuclear protein
NF: neurofilaments
PCr: phosphocreatine
pHi: intracellular pH
Pi: inorganic phosphate
PTIBS: Porcine Thoracic Injury Behavior Scale
PRISMA: Preferred Reporting Items for Systematic Reviews and Meta-Analyses
SCBF: spinal cord blood flow
SCI: spinal cord injury
SSEP: somatosensory evoked potential
TNFα: tumor necrosis factor-alpha
