# Supplementary material for: Porcine Model of Spinal Cord Injury: A Systematic Review
Source: Neurotrauma Rep. 2022 Sep 1;3(1):352–68. doi: 10.1089/neur.2022.0038 (PMC9531891; doi:10.1089/neur.2022.0038)
Supplement: Supplemental data [file Supp_TableS1.docx]

**Supplementary Table 1** Search queries for systematic review.

| Database | Search Algorithm |
| --- | --- |
| PubMed | (“Spinal cord injuries”[mesh] OR “Spinal Cord Injur*”[tw] OR “Spinal Cord Injury”[tw] OR “SCI”[tw]) AND (“Swine”[mesh] OR “Swine”[tw] OR “Pig*”[tw] OR “Minipig*”[tw] OR “Porcine”[tw]) AND (“Models, Animal”[mesh] OR “Disease Models, Animal”[mesh] OR “Animal Model*”[tw] OR “Pig Model*”[tw] OR “Swine Model*”[tw] OR “Porcine Model*”[tw] OR “Disease Model*”[tw] OR “Model*”[tw]) |
| Embase | ('Spinal cord injury'/exp OR 'Spinal Cord Injur*':ti,ab,kw OR 'Spinal Cord Injury':ti,ab,kw OR 'SCI':ti,ab,kw ) AND (‘Pig'/exp OR 'Swine':ti,ab,kw OR 'Pig*':ti,ab,kw OR 'Minipig*':ti,ab,kw OR 'Porcine':ti,ab,kw ) AND ('Animal Model'/exp OR 'Porcine Model'/exp OR 'Disease Model'/exp OR ‘Model’/exp OR 'Animal Model*':ti,ab,kw OR 'Disease Model*':ti,ab,kw OR 'Model*':ti,ab,kw OR 'Pig Model*':ti,ab,kw OR 'Swine Model*':ti,ab,kw OR 'Porcine Model*':ti,ab,kw) |
| Cochrane Library | ([mh “Spinal cord injuries”] OR “Spinal Cord Injur*” OR “Spinal Cord Injury” OR “SCI”) AND ([mh “Swine”] OR “Swine” OR “Pig*” OR “Minipig*” OR “Porcine”) AND ([mh “Models, Animal”] OR [mh “Disease Models, Animal”] OR “Animal Model*” OR “Pig Model*” OR “Swine Model*” OR “Porcine Model*” OR “Disease Model*” OR “Model*”) |
| Scopus | TITLE-ABS-KEY(“Spinal cord injuries” OR “Spinal Cord Injur*” OR “Spinal Cord Injury” OR “SCI”) AND TITLE-ABS-KEY(“Swine” OR “Pig*” OR “Minipig*” OR “Porcine”) AND TITLE-ABS-KEY(“Animal Model*” OR “Pig Model*” OR “Swine Model*” OR “Porcine Model*” OR “Disease Model*” OR “Model*”) |
