# Supplementary material for: Porcine Model of Spinal Cord Injury: A Systematic Review
Source: Neurotrauma Rep. 2022 Sep 1;3(1):352–68. doi: 10.1089/neur.2022.0038 (PMC9531891; doi:10.1089/neur.2022.0038)
Supplement: Supplemental data [file Supp_TableS2.docx]

**Supplementary Table 2:** SYRCLE’s risk of bias analysis of studies that included an intervention.

| **Lead author** | **Year** | **Was the allocation sequence adequately generated and applied? (*)** | **Were the groups similar at baseline or were they adjusted for confounders in the analysis?** | **Was the allocation adequately concealed? (*)** | **Were the animals randomly housed during the experiment?** | **Were the caregivers and/or investigators blinded from knowledge which intervention each animal received during the experiment?** | **Were animals selected at random for outcome assessment?** | **Was the outcome assessor blinded?** | **Were incomplete outcome data adequately addressed? (*)** | **Are reports of the study free of selective outcome reporting? (*)** | **Was the study apparently free of other problems that could result in high risk of bias? (*)** |
| --- | --- | --- | --- | --- | --- | --- | --- | --- | --- | --- | --- |
| Bernards | 2006 | Y | Unclear | Y | Unclear | Unclear | NA | Unclear | NA | Y | Y |
| Cheung, Streijger | 2020 | Y | Unclear | Y | Unclear | Unclear | NA | Unclear | NA | Y | Y |
| Fadeev | 2020 | Unclear | Unclear | Unclear | Unclear | Unclear | NA | Unclear | NA | Y | Y |
| Gao | 2019 | Unclear | Unclear | Unclear | Unclear | Unclear | NA | Unclear | NA | Y | Y |
| Gedrova | 2018 | Y | Y | Y | Unclear | Unclear | NA | Unclear | Y | Y | Y |
| Guest | 2018 | Y | Unclear | Y | Unclear | Unclear | Y | Y | Y | Y | Y |
| Hachmann | 2013 | Unclear | Unclear | Unclear | Unclear | Unclear | NA | Unclear | NA | Y | Y |
| Islamov | 2020 | Unclear | Y | Unclear | Unclear | Unclear | NA | Y | NA | Y | Y |
| Islamov | 2021 | Unclear | Unclear | Unclear | Unclear | Unclear | NA | Unclear | NA | Y | Y |
| Islamov | 2017 | Unclear | Unclear | Unclear | Unclear | Unclear | NA | Unclear | NA | Y | Y |
| Keller | 2020 | Y | Unclear | Y | Unclear | Unclear | NA | Y | Y | Y | Y |
| Kowalski | 2016 | Unclear | Unclear | Unclear | Unclear | Unclear | NA | Unclear | NA | Y | Y |
| Lim | 2010 | Unclear | Unclear | Unclear | Unclear | Unclear | NA | Unclear | NA | Y | Y |
| Martirosyan | 2015 | Unclear | Unclear | Unclear | Unclear | Unclear | NA | Unclear | NA | Y | Y |
| Modi | 2011 | Unclear | Unclear | Unclear | Unclear | Unclear | NA | Unclear | Y | Y | Y |
| Mukhamedshina | 2019 | Y | Unclear | Y | Unclear | Unclear | Y | Y | NA | Y | Y |
| Sarwahi | 2020 | Unclear | Unclear | Unclear | Unclear | Unclear | Y | Unclear | NA | Y | Y |
| Shadgan | 2019 | Unclear | Unclear | Unclear | Unclear | Unclear | NA | Unclear | NA | Y | Y |
| Shadgan | 2018 | Unclear | Unclear | Unclear | Unclear | Unclear | NA | Unclear | NA | Y | Y |
| Shulman | 2021 | Unclear | Unclear | Unclear | Unclear | Unclear | NA | Y | NA | Y | Y |
| Solis | 2013 | Y | Unclear | Y | Unclear | Unclear | NA | Y | NA | Y | Y |
| Streijger | 2021 | Y | Unclear | Y | Unclear | Unclear | NA | Y | NA | Y | Y |
| Streijger | 2015 | Y | Unclear | Y | Unclear | Unclear | Unclear | Unclear | NA | Y | Y |
| Streijger | 2016 | Unclear | Unclear | Unclear | Unclear | Unclear | NA | Unclear | Y | Y | Y |
| Streijger | 2016 | Y | Unclear | Y | Unclear | Unclear | NA | Y | NA | Y | Y |
| Streijger | 2018 | Y | Unclear | Y | Unclear | N | NA | Y | Y | Y | Y |
| Williams | 2020 | Y | Unclear | Y | Unclear | Y | NA | Unclear | Y | Y | Y |
| Zavodska | 2018 | Unclear | Unclear | Unclear | Unclear | Unclear | Y | Y | NA | Y | Y |
| Zurita | 2013 | Y | Unclear | Y | Unclear | Unclear | Y | Unclear | NA | Y | Y |
| Zurita | 2008 | Y | Unclear | Y | Unclear | Unclear | NA | Unclear | NA | Y | Y |
