# Supplementary material for: Porcine Model of Spinal Cord Injury: A Systematic Review
Source: Neurotrauma Rep. 2022 Sep 1;3(1):352–68. doi: 10.1089/neur.2022.0038 (PMC9531891; doi:10.1089/neur.2022.0038)
Supplement: Supplemental data [file Supp_TableS3.docx]

**Supplementary Table 3:** Summary of the study characteristics for studies that did not include an intervention.

| **Lead author** | **Year** | **Breed** | **N** | **Weight (kg)** | **Injury location** | **Cause of injury** | **Time to endpoint (categories)** | **Conclusion** |
| --- | --- | --- | --- | --- | --- | --- | --- | --- |
| Akino | 1997 | NS | 12 | 15-25 | L2 | 20 g weight drop from 20 cm | <1 day | In vivo spectra of injured cords revealed a reduction in adenosine triphosphate, phosphocreatine, and intracellular pH, and an increase in inorganic phosphate. |
| Chen | 2011 | NS | 12 | 32-39 | L1-L2 | Gunshot wound via 79 machine gun at 200 m distance or 40 g weight drop from 12.5 cm | other | Gunshot led to animal death within 5.2-7.5 hrs likely from hypovolemic shock and organ damage in the heart and kidneys. |
| Cheung, Tu | 2020 | Yucatan | 6 | 25-31 | T10 | 50 g weight drop from 16.73 cm followed by 100 g compression for 30 mins | <1 week | A near-infrared spectroscopy sensor provided real-time measurements of spinal cord oxygenation and hemodynamics that correlated with invasive intraparenchymal measures. |
| Del Cerro | 2021 | Other | 8 | 10-15 | C6 | Hemisection | <3 mo | Hemisection produced a chronic, incomplete SCI with permanent impairment of the right frontal limb with gradual recovery of the other 3 legs. |
| Foditsch | 2018 | Gottingen | 10 | 21-26 | T12 | Balloon catheter at 2 atm for 30 mins | <6 mo | A minimally invasive model of CT-guided SCI with a puncture needle and guide wire produced stable severe neurological deficits. |
| Foditsch | 2017 | Gottingen | 7 | 18-30 | T12-T13 | Transection via bipolar electrocautery | <6 mo | Following SCI, there were smaller bladder volumes, increased incidence of incontinence, no urinary tract infections, loss of smooth muscle tissue, and increased connective tissue. |
| Huang | 2013 | Domestic | 6 | 15-16 | T9-T12 | 50 g weight drop from 15 cm | <1 week | Contrast-enhanced ultrasound demonstrated hyperperfusion adjacent to the injury site and hypoperfusion at the epicenter. |
| Jones, Cripton | 2012 | Yucatan | 13 | NS | T11 | 20 or 100 g weight drop from 32 or 125 cm followed by 100 g compression for 8 hours | non-survival | Animals with a moderate injury exhibited gradual swelling and spinal cord deformation that resolved after several hours, while those with high-severity injury showed immediate swelling that lasted for several hours. |
| Jones | 2013 | Yucatan | 14 | 20 | T10-T11 | 20 g weight drop from 32 cm or 125 cm followed by 100 g compression for 8 hours | non-survival | Injury severity affects peak CSF pressure and pressure impulse during SCI. |
| Jones, Lee | 2012 | Yucatan | 17 | 22.5 ± 2.0 | T10 | 50 or 100 g weight drop from 50 cm | non-survival | Fiber optic pressure transducers can be used to measure CSF pressure. Heavier weight drops caused more negative peak pressure. |
| Jones, Newell | 2012 | Yucatan | 14 | 20-22 | T11 | 20 g weight drop from 32 or 125 cm followed by 100 g compression for 8 hours | non-survival | Thecal occlusion after SCI resulted in higher cranial than caudal CSF pressure, suggesting that relying on lumbar catheter CSF measurements for drainage protocols may not reliably affect spinal cord perfusion pressure in a state of compression. |
| Jutzeler | 2018 | Yucatan | 12 | 18.5-25 | T10 | 50 g weight drop from 20 com followed by 100 g compression for 5 mins | other | Sensory and motor responses in forelimbs progressively increased after SCI and is similar to the observed response in humans, illustrating cortical reorganization after SCI. |
| Keung | 2021 | Yucatan | 40 | 21-44 | T2 or T10 | 50 g weight drop from 16-20 cm followed by 100 g compression for 5 mins | <6 mo | Porcine SCI model can be used to study lower urinary tract dysfunction after SCI. |
| Kim | 2017 | Yucatan | 1 | 60 | NS | 50 g weight drop from 20 cm followed by compression for 5 mins | <1 week | A SCI modeling system using a multimodal sensor measured the SCI impact parameters of impact velocity, impulsive force, and dural displacement. |
| Kim | 2019 | Yucatan | 7 | 19.5-34.0 | T10 | 50 weight drop from 20 cm followed by 150 g compression for 5 mins | <3 mo | Measured biomechanical impact parameters did not correlate with the histological or behavioral measure, while the dorsoventral diameter of the dural sac and ventral CSF space were strong predictors of the measures. |
| Kuluz | 2010 | Multiple | 14 | 5 - 7 | T7 | 6 mm diameter impactor with depth of either 5/8 mm and 60/80 psi for complete SCI and 3mm and 30 psi for incomplete SCI | <1 mo | Complete SCI led to larger volumes of necrotic tissue and less functional recovery compared to incomplete SCI. MRI was performed safely and showed injury well. |
| Lee | 2013 | Yucatan | 41 | 20-25 | T10-T11 | 50 g weight drop from varying heights (5, 10, 20, 30, 40, 50 cm) followed by 150 g compression for 5 mins | <3 mo | Animals with a more severe injury had less spared white and gray matter and less neurofilament immunoreactivity. PTIBS scores correlated strongly with the extent of tissue sparing at the site of injury. |
| Montes | 2017 | Domestic | 5 | 29 - 39 | T8-T9 | Compression by two sticks placed on both sides of cord and brought together | non-survival | Increasing latency and decreasing amplitude of the evoked potentials were observed after a mean progressive displacement of the sticks of 3.2 ± 0.9 mm. The potential returned after compression removal (16.8 ± 3.2 min). |
| Navarro | 2012 | Gottingen | 21 | 18-23 | T12 | Computer controlled impactor with force of 1.5, 2, or 2.5 kg at 3 cm/sec | <1 yr | 1.5 kg, 2 kg, and 2.5 kg force showed normal ambulation 10 days after injury, progressive partial neurological recovery, and complete loss of motor and sensory function, respectively. |
| Okon | 2013 | Yorkshire | 16 | 25-35 | T10 | 50 g weight drop from 50 cm followed by 150 g compression for 5 mins or 4 hrs | < 1 day | Contusion with sustained compression produced a prolonged and dramatic increase in the lactate-pyruvate ratio as a marker of tissue hypoxia, while contusion injury alone let to a transient and less significant elevation of the L/P ratio. |
| Ruggiero | 1997 | Sus Scrofa | 8 | NS | C1 | Transection | < 1 day | Following transection, fos-like immunoreactivity (FLI) was induced in spinal laminae I, V, VII and X and the intermediolateral cell column but not in sensory ganglia. |
| Santamaria | 2019 | Yucatan | 52 | 17 - 20 | T9 | Impactor with 3.5 (for 30 psi) or 2.35 (for 50 and 60 psi) mm compression depth, 30, 50, or 60 psi chamber pressure, 150 msec dwell time | <3 mo | Early MRI and ultrasound data strongly correlated to locomotor outcomes. |
| Skinner | 2013 | Domestic | 3 | 36 - 44 | Thoracic | Electrocautery (35 W) delivered within 6-8 mm of the spinal cord | non-survival | Electrocautery can lead to thermally generated SCI. EMG recordings may be used to detect thermal injury to the spinal cord before MEP loss. |
| Skinner | 2009 | NS | 12 | NS | Thoracic | Distraction, extradural compression with metal calipers, and compression with spring loaded clips | non-survival | EMG injury discharges preceded and may anticipate transcranial MEP loss. |
| Skinnider | 2021 | Yucatan | 43 | 20-30 | T10 | 50 g weight drop from 40, 20, or 10 cm followed by 150 g compression for 5 mins | <3 mo | CSF levels of GFAP at 24 hr were correlated to both baseline injury severity and hindlimb neurological recovery in pigs, and therefore can be used to monitor injury progression and therapeutic response in animal studies. |
| Streijger | 2017 | Yucatan | 8 | 23 ± 0.5 | T10 | 50 g weight drop from 20 cm followed by 150 g compression for 60 mins | <1 week | Traumatic SCI resulted in an expanding area of ischemia/hypoxia, with ongoing physiological perturbations up to 7 days post-injury. |
| Tigchelaar | 2017 | Yucatan | 16 | 20 - 30 | T10 | 50 g weight drop from 10, 20, or 40 cm followed by 150 g compression 5 mins | <3 mo | Serum miRNA expression levels at 1- and 3-days post injury strongly correlated with outcome measures at 12 weeks post injury |
| Wang | 1996 | NS | 16 | 51-71 | T12 or L1 | Rifle with spherical steel balls (2.47 and 4.58 g) or natural fragments (3.2-5.1 g) shot at 920-1005 m/s | <1 day | High velocity missiles led to severe paraplegia of the hind legs with diffuse hemorrhage and tissue damage in the spinal cord. |
| West | 2020 | Multiple | 15 | 25-31 for Yorkshire; 26-36 for Yucatan) | T2 or T10 | 50 g weight drop from 20 cm followed by 150 or 100 g compression for 5 mins or 2 hrs | other | After T2 SCI, SBP, MAP and HR increased acutely and subsequently decreased, while after T10-SCI, the hemodynamic indices remained largely unaffected. |
| Zahra | 2010 | Other | 15 | 5-9 | C3-C4 | 8 mm diameter impactor with depth of 5 mm and pressure of 80 psi for 0.3s | <1 day | Following SCI, tachycardia and hypotension developed with decreased systemic and pulmonary vascular resistance. There was an increase in cardiac output, sustained initially by an increase in heart rate and maintained by an increase in stroke volume. |
| Züchner | 2021 | Other | 8 | 20-25 & 20 | T11-T12 | Spring-load impactor with force of 12.5 N followed by 7.3 N compression for 3 mins | <3 mo | Different impact parameters, including impact force, static compression force, and compression time, created different levels of injury severity. |
| Züchner | 2019 | Domestic | 20 | 25-50 | T10-T11 for weight drop T12-T13 for spring-load | 50 g weight drop from 24, 34, and 44 cm; spring-loaded impactor with force of 45.9N followed by compression for 4 mins | non-survival | An apparatus can perform biomechanically reproducible SCI in large animals. Evaluation of the injury using neurophysiological recordings, MRI scans, and histology showed consistency between impacts. |
| Zurita | 2012 | Other | 20 | 15-20 | T12-L1 | Two surgical Heifetz’s clips for 30 mins | <1 yr | Healthy paraplegic minipigs can be maintained for at least a year following SCI. |
