# Supplementary material for: Porcine Model of Spinal Cord Injury: A Systematic Review
Source: Neurotrauma Rep. 2022 Sep 1;3(1):352–68. doi: 10.1089/neur.2022.0038 (PMC9531891; doi:10.1089/neur.2022.0038)
Supplement: Supplemental data [file Supp_TableS4.docx]

**Supplementary Table 4:** Summary of the study characteristics for studies that tested interventions.

| **Lead author** | **Year** | **Breed** | **N** | **Weight (kg)** | **Injury location** | **Cause of injury** | **Time to endpoint** | **Intervention** | **Conclusion** |
| --- | --- | --- | --- | --- | --- | --- | --- | --- | --- |
| Bernards | 2006 | NS | NS | 18-22 | T13 | 25 g weight drop from 45 cm | non-survival | Intravenous or intrathecal methylprednisolone 30 min post-SCI | There is no effect of post-op methylprednisolone following SCI. |
| Cheung, Streijger | 2020 | Yucatan | 24 | 21-30 | T10 | 50 g weight-drop from 20 cm followed by 100 g compression for 2 hrs | non-survival | Norepinephrine administered during compression, after decompression, or both | MAP augmentation following SCI increases spinal cord blood flow and oxygenation, but the risk of intraparenchymal pressure and hemorrhage may increase with infusion time. |
| Fadeev | 2020 | Vietnamese | 6 | 15-20 | T8-T9 | 50 g weight drop from 50 cm | <3 mo | Motor rehabilitation on a treadmill with epidural electrical stimulation at C5 and L2 | Epidural electrical stimulation following SCI improves behavioral outcomes and restores M-response and H-reflex in MEPs. |
| Gao | 2019 | Yucatan | 2 | 20-25 | T10 | 50 g weight drop from 20 cm followed by 150 g compression for 5 mins | <1 day | Nanoparticles (Poly (d,l-lactide co-glycolide, PLGA)-based) loaded with a near-infrared dye | Nanoparticles are more likely to localize to the SCI lesion site than the uninjured tissue. |
| Gedrova | 2018 | Gottingen | 30 | 25-35 | L3 | Impactor with 8N or 15N force delivered at 30 mm/sec | <3 mo | Hypothermia (4°C saline or DMEM culture medium) applied 30 min after SCI at the injury site for 5 h via a perfusion chamber | Delivering saline hypothermia to the injury site after a more mild SCI improves hind limb function and ability to walk at 9 weeks compared to non-treated animals. |
| Guest | 2018 | Gottingen | 4 | 25 | T10 | 50 g weight drop from 40 cm followed by 150 g compression for 5 mins | <1 day | Pial opening, removal of necrotic tissue, and implantation of PLGA-PLL scaffold at the site of injury 4, 6, or 24 hr after injury | Insertion of a PLGA-PLL scaffold is feasible. |
| Hachmann | 2013 | Domestic | 6 | 25-35 | T4 | Complete transection | non-survival | Epidural stimulation around L5 or intraspinal microstimulation around L2. | Epidural and intraspinal stimulation can evoke extension, flexion, adduction, and abduction of the knee and hip joints, with the joint angle change increasing proportionally to stimulation amplitude. |
| Islamov | 2020 | Vietnamese | 6 | 20-25 | T8-T9 | 50 g weight drop from 50 cm | <3 mo | Locomotor training facilitated with epidural electrical stimulation (EES) and cell-mediated triple gene therapy with umbilical cord blood mononuclear cells overexpressing recombinant vascular endothelial growth factor, glial-derived neurotrophic factor, and neural cell adhesion molecule. | A combination of EES and *ex vivo* triple gene therapy improves locomotor performance and promotes positive remodeling of the spinal cord following SCI. |
| Islamov | 2021 | Vietnamese | 3 | 30 | T8-T9 | 50 g weight drop from 50 cm | <1 week | Infusion of leucoconcentrate obtained from pig peripheral blood transduced with a chimeric adenoviral vector carrying eGFP 4 hrs after surgery. | Leucocyte transduction and their migratory potential can be used for the production of biologically active molecules to correct pathological conditions. |
| Islamov | 2017 | Vietnamese | 2 | 5 | T9-T10 | 50 g weight drop from 50 cm | <3 mo | Intrathecal injection at L4-L5 of genetically engineered UCBC 10 days after injury. | Treatment with genetically engineered UCBC following SCI leads to significant histological, electrophysiological, and clinical improvements. |
| Keller | 2020 | Gottingen | 11 | 21-26 | T11-T12 | Balloon catheter at 2 atm for 30 mins | <6 mo | Sacral neuromodulation or pudendal neuromodulation 1 week post-SCI using electrodes and pacemaker. | Sacral neuromodulation after SCI improves bladder function and reduces scarring more than pudendal neuromodulation or untreated pigs. |
| Kowalski | 2016 | Other | 10 | 22.3 ± 0.8 | T6 | Epidural application of two surgical Heifetz's clips for 30 mins | <3 mo | Epidural electrical stimulation at T9-T12 beginning10-14 days post-injury and continuing daily for three months. | Electrical spinal cord stimulation to restore expiratory muscle function following SCI can be safe and useful. |
| Lim | 2010 | Yucatan | 5 | 29.5 ± 9.48 | L7-S1 | Transection using tenotomy scissors | <1 mo | Injection of GFP-pNSC cells suspended in artificial CSF at the spinal cord defect. | Yucatan somatic cell nuclear transfer (SCNT) clones can be used to develop a SCI model for cellular transplantation research. |
| Martirosyan | 2015 | Yorkshire | 15 | NS | T5 | 20 g weight drop from 15 cm | non-survival | MAP elevation via phenylephrine drip, CSFD open at 5 mmHg, or both one hour after SCI. | Following SCI, MAP elevation with CSFD can increase SCBF more than MAP elevation alone. |
| Modi | 2011 | Other | 10 | 51.9 ±1 | T11-T12 | T11 and T12 vertebrectomy | non-survival | Spinal shortening. | Spinal shortening of a certain height can lead to a loss of MEPs, SCBF, and limb movement. |
| Mukhamedshina | 2019 | Vietnamese | 17 | 9-12 | T10 | 50 g weight drop from 20 cm followed by compression for 10 mins | <6 mo | Application of AD-MSCs on top of the injury at 6 weeks. | Delivery of allogenic MSCs derived from adipose tissue (AD-MSCs) shows some functional improvement and restoration of SEPs, without improving conductivity along the lateral column. |
| Sarwahi | 2020 | Sus Scrofa | 14 | 33-73 | Thoracic | balloon inflation of 0.25 cm^3 every 10 mins | non-survival | MAP augmentation via phenylephrine and blood volume expansion via hetastarch. | SCBF can be used to precipitate MEP loss. |
| Shadgan | 2019 | Yorkshire | 9 | 25-30 | T10 | 50 g weight drop from 50 cm followed by 150 compression for 30 mins | non-survival | Episodes of ventilatory hypoxia and alterations in MAP via norepinephrine and nitroprusside. | A multi-wavelength near-infrared spectroscopy sensor can detect and measure real-time changes in spinal cord oxygenation following SCI. |
| Shadgan | 2018 | Yorkshire | 6 | 25 | T10 | 50 g weight drop from 50 cm followed by 150 g compression for 30 mins | non-survival | MAP pressure alterations. | Elevation of MAP following SCI can reduce spinal cord tissue damage as observed by a significant increase in spinal cord tissue mitochondrial cytochrome aa3 concentration as measured by a near-infrared spectroscopy sensor. |
| Shulman | 2021 | Vietnamese | 12 | 8 | T10 | 50 g weight drop from 20 cm followed by compression for 10 mins | <6 mo | Application of PBMCs embedded in a fibrin matrix on top of the injury at 6 weeks after injury. | PBMCs delivered after SCI can increase tissue integrity adjacent to the injury epicenter and partially restore conduction along the posterior column. |
| Solis | 2013 | Yucatan | 6 | 58.5 ± 7.4 | L2 | Hemisection | <3 mo | Loading paralyzed limbs with 25% of body weight 4h/day for 4 consecutive day each week for one month with or without concurrent IES. | Intermittent electrical stimulation may be an effective technique for preventing the formation of deep tissue injury in loaded muscles after SCI. |
| Streijger | 2021 | Yucatan | 35 | 18.5-34.5 | T10 | 50 g weight drop from 20 cm followed by 150 g compression for 5 mins | other | Expansion duraplasty, using biological dural substitute, either before or after SCI. | Duraplasty following SCI does not provide any clear evidence of long-term behavioral or tissue sparing. |
| Streijger | 2015 | Yucatan | 32 | 20-30 | T10 | 50 g weight drop from 20 cm followed by 150 g compression for 5 mins | other | Animals were subjected to resonance vibration (5.7 ± 0.46 Hz) for either 1.5 or 3 h 30 mins after inducing SCI | Resonance vibration exposure following SCI is not detrimental to functional or histological outcomes. |
| Streijger | 2016 | Yucatan | 33 | 20-30 | T10 | 50 g weight drop from 20 cm followed by 150 g compression for 5 mins | <3 mo | Exposure to vibrations simulating helicopter (3 h at 17 Hz 30 min post injury) or MRAP transport (3.5 h at 2 Hz 1 hr post injury). | Exposures to vibration from helicopter or mine-resistant ambush-protected vehicle does not significantly impair functional outcome following SCI. |
| Streijger | 2016 | Yucatan | 22 | 22.19 ± 0.28 | T10 | 50 g weight drop from 20 cm followed by 150 g compression for 5 mins | <3 mo | Infusion of AC105, magnesium sulfate (MgSO4), or saline following injury with 4 additional infusions every 6 hrs. | Treatment with a polyethylene glycol (PEG) formulation, AC105, or MgSO4 does not result in improvements in locomotor recovery. |
| Streijger | 2018 | Yorkshire | 26 | 25-32 | T10 | 50 g weight drop from 20 cm followed by 150 g compression for 3 hrs | <1 day | MAP elevation of 20 mmHg via NE, PE, or DA or after 3 hr of compression. | Norepinephrine promotes better restoration of blood flow and oxygenation than phenylephrine. Both are associated with a gradual decrease in the lactate to pyruvate (L/P) ratio after decompression. |
| Williams | 2020 | Yucatan | 32 | 20-25 | T2 | 50 g weight drop from 16 cm followed by 150 g compression for 2 hrs | other | Augment LV contractility via B-agonist dobutamine or norepinephrine given 30 mins post-SCI. | Augmenting LV contractility with a β-agonist, dobutamine, following SCI increases SCO_2_ and SCBF more effectively than vasopressor therapy. |
| Zavodska | 2018 | Gottingen | 24 | 25-35 | L3 | Computer controlled 5 mm diameter impactor with force of 18N | <3 mo | Deliver local hypothermia 30 min post injury 5 hr. | Delivery of local hypothermia, at either 15 or 24°C, does not result in significant improvements of neurological status. |
| Zurita | 2013 | Other | 5 | 18-20 | L2-L3 | Two surgical Heifetz’s clips for 30 mins | <1 yr | Intrathecal administration of BMSC suspended in autologous plasma 3 mo after injury. | Bone marrow stromal cells delivered after SCI, can lead to clinical improvement, recovery of somatosensory evoked potentials, and spinal cord regeneration following injury. |
| Zurita | 2008 | Other | 10 | 20 | T12 - T13 | Two surgical Heifetz’s clips for 30 mins | <6 mo | Injection of autologous BMSC into lesion zone and adjacent subarachnoid space 3 mo after injury. | Bone marrow stromal cells delivered after SCI can express neuronal or glial markers and lead to progressive functional recovery following injury. |
